# Spontaneous rupture of giant gastric stromal tumor into gastric lumen

**DOI:** 10.1186/1477-7819-3-11

**Published:** 2005-02-15

**Authors:** Rajiv M Mehta, Vayoth O Sudheer, Anil K John, Raghavan R Nandakumar, Puneet S Dhar, Surendran Sudhindran, Vallath Balakrishnan

**Affiliations:** 1 Department of Gastroenterology, Amrita Institute of Medical Sciences, Cochin, Kerala, 642026 India; 2 Department of Surgical Gastroenterology, Amrita Institute of Medical Sciences, Cochin, Kerala, 642026 India

## Abstract

**Background:**

Gastrointestinal stromal tumors (GIST) constitute a large majority of mesenchymal tumors of the gastrointestinal (GI) tract, which express the c-kit proto-oncogene protein, a cell membrane receptor with tyrosine kinase activity. GI stromal tumors of the stomach are usually associated with bleeding, abdominal pain or a palpable mass.

**Case presentation:**

A 75-year-old male presented with upper abdominal pain and palpable mass. Computed tomographic (CT) scan of the abdomen showed a large mass arising in the posterior aspect of fundus, body, and greater curvature of the stomach. Second day after the admission, there was significant reduction in the size of the tumor, clinically as well as radiologically. Endoscopic biopsy showed large bulge in fundus and corpus of the stomach posteriorly with an opening in the posterior part of the corpus, and biopsy from the edge of the opening reveled GIST. Patient underwent curative resection.

**Conclusion:**

Spontaneous ruptured of giant gastric stromal tumor is very rare presentation of stomach GIST. Thorough clinical examination and timely investigation can diagnose rare complication.

## Background

Gastrointestinal stromal tumors (GIST) are the most common form of mesenchymal tumors arising from the gastrointestinal (GI) wall, mesentery, omentum or retroperitoneum that express the c-kit proto-oncogene protein [1]. This expression of c-kit distinguishes GIST from true leiomyomas, leiomyosarcomas, and other mesenchymal tumors of the GI tract [1,2]. Stomach (60–70%) and small intestine (20–30%) is the most common site for GIST [2]. Approximately 10–30% of patients with GIST may be asymptomatic. Stomach and small intestinal stromal tumors are usually associated with abdominal pain, GI bleeding or palpable mass. Around 30% of all GISTs are malignant and liver is the most common site for metastasis. Surgical resection is the primary treatment of GIST [3]. The 5-year survival following curative resection ranges from 20–80% [3-7]. Imatinib mesylate, tyrosine kinase inhibitor, is the first effective drug with response rate of 54% in the treatment of metastatic GIST. We report here a case of GIST which presented with rupture in to the gastric lumen.

## Case presentation

A 75-year-old diabetic male presented with dull upper abdominal pain of one-week duration. He noticed swelling in left upper abdomen. There was no history of vomiting, fever or gastrointestinal bleeding. He had no significant medical or family history and was non-smoker and non-alcoholic. Physical examination showed a 14 × 10 cm mass palpable in epigastrium and left hypochondrium with minimal intrinsic mobility.

**Figure 1:**
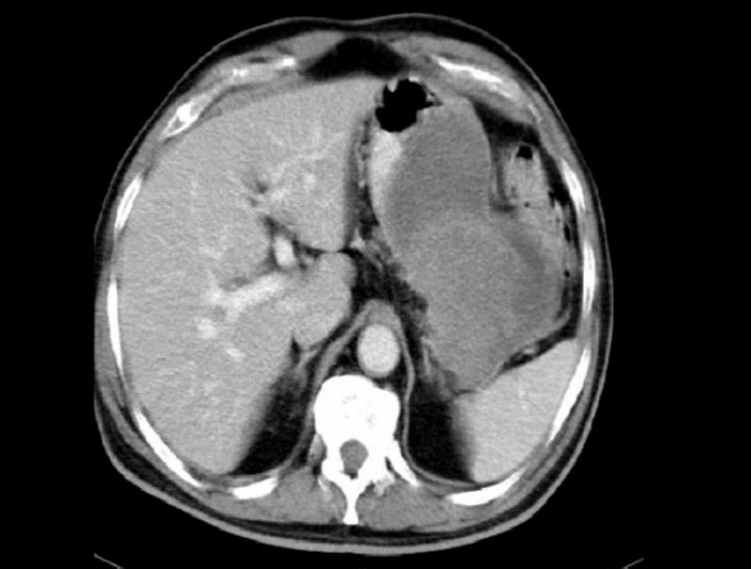
**CT**-Scan showed large heterogeneous mass of 13 × 10 cm size extending from the tail of pancreas to anterior prararenal space, lesser sac to gastrosplenic ligament enveloping the posterior aspect of fundus, body and greater curvature.

**Figure 2:**
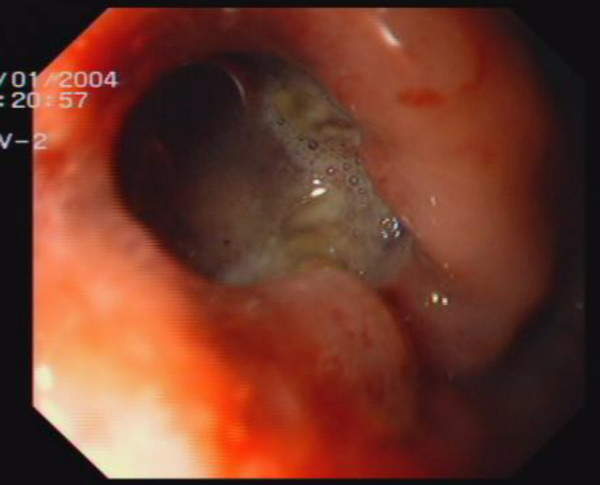
Upper endoscopy picture shows opening in the posterior part of the corpus with edematous margin with dissemination of serous fluid and necrotic material in to the stomach.

**Figure 3:**
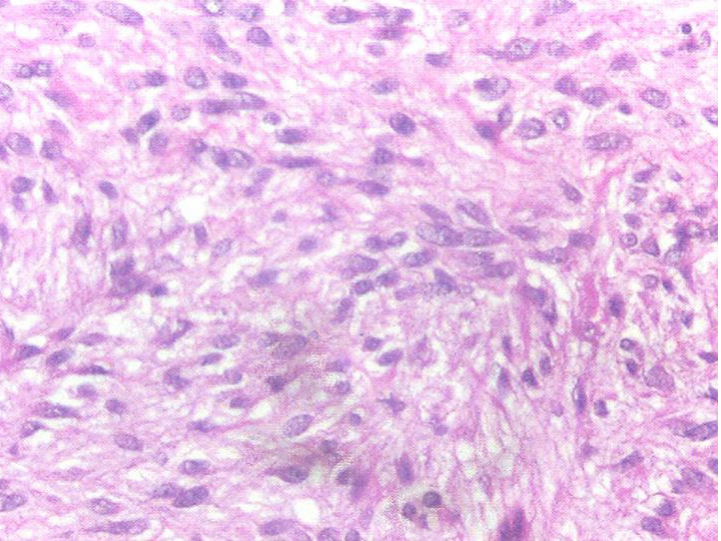
Photomicrograph showing upper endoscopy biopsy specimen of gastric GIST showing multiple spindle cells with eosinophilic cytoplasm and ovoid to elongated nuclei

**Figure 4:**
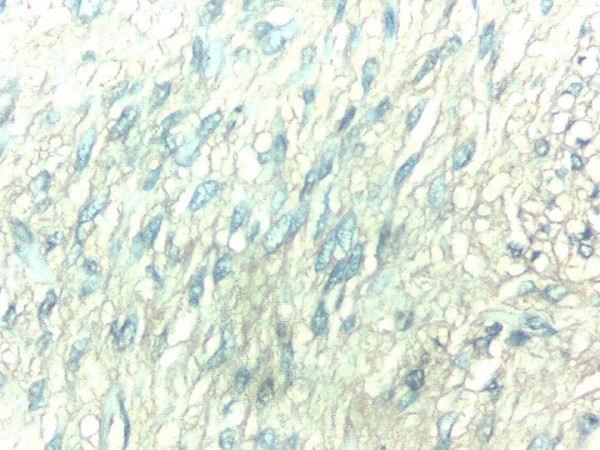
Photomicrograph of biopsy specimen with immunohistochemical staining for CD117

Routine biochemical investigations were normal. Ultrasonogram and CT-scan of the abdomen showed large heterogeneous mass of 13 × 10 cm extending from the tail of pancreas to anterior pararenal space, lesser sac to gastrosplenic ligament enveloping the posterior aspect of fundus, body and greater curvature (Figure 1). One day after the admission, examination showed reduction in the size of palpable mass to 8 × 6 cm size which was not associated with aggravation of the symptoms. Ultrasonography of the abdomen was repeated which showed reduction in the diameter of mass to 8 × 8 cm. Upper endoscopy showed large bulge in fundus and corpus of the stomach posteriorly with an opening in the posterior part of the corpus with edematous margin with dissemination of serous fluid and necrotic material in to the stomach (Figure 2). Fluid analysis was normal for CEA and CA 19-9. Biopsy taken from the edge of the opening showed bundles of spindle cells with elongated nuclei and tumor cells (Figure 3) and was strongly positive for CD117 immunohistochemical examination, diagnostic of gastrointestinal stromal tumor (Figure 4). At laparotomy a large tumor was seen arising from the posterior wall of stomach measuring 8 × 8 cm, which has ruptured into the gastric lumen, and was infiltrating the upper pole of spleen, anterior capsule of pancreas and mesocolon. He underwent total gastrectomy and splenectomy with esophagojejunostomy, and segmental transverse colectomy. Histopathology of resected specimen showed large spindle cell GIST with >5/50 HPF (high-power field) mitotic activity. Postoperative period was uneventful. Postoperatively he was put on imatinib mesylate 400 mg once daily. Patient is asymptomatic on follow up for 11 months.

## Discussion

GI stromal tumors express c-kit protein also known as CD 117, and is considered as highly specific marker that differentiates GIST from other mesenchymal tumors such as leiomyomas [8-10]. The majority of GISTs occur in the stomach (60–70%) and small intestine (20–30%) [9]. GIST arises from the stomach, presented with abdominal pain, GI bleeding or palpable mass. Around 20–30% of GISTs detected during surgery for intestinal obstruction or bleeding [9]. Among the diverse clinical presentation of stomach GISTs, spontaneous ruptured in to peritoneal cavity lead to peritonitis [11], extragastric growth [12], complicated with hiatus hernia [13], ruptured of gastric stromal tumor with cystic degeneration presenting as hemoperitoneum [14], gastric stromal tumor with myxoid degeneration [15] have been reported in the literature. Our patient had giant gastric GIST, which ruptured into the stomach with dissemination of its necrotic tissue in the stomach. There was no aggravation of the symptoms. Abdominal examination revealed reduction of the size of tumor from 13 × 10 cm to 8 × 6 cm size. Ultrasound abdomen also confirmed 40% reduction in the size of the tumor. Upper endoscopy biopsy from the edge of the opening, posterior wall of the stomach was suggestive of GIST. Since most of the GISTs are submucosal and grow endophytically, preoperative tissue diagnosis is difficult. In our patient biopsy from the edge of the rent and deep inside the opening confirmed diagnosis of GIST preoperatively. Patient underwent complete tumor resection, with uneventful postoperative period.

Assessment of malignant potential of a primary GIST lesion is difficult in many cases; even as small as less than 2 cm size GIST has certain malignant potentials [9]. Currently, there is no single prognostic factor that can be used alone to predict tumor behavior. Biological behavior of tumor also depends on the location; for example, GISTs arising from the small bowel or colon are generally associated with a less favorable outcome than those arising in the stomach [16]. Radiological and surgical factors that have been used to determine malignancy include invasion to adjacent organs; omental or peritoneal seeding; tumor recurrence after surgical resection; or distant metastasis [8,17]. Pathological factors that determine malignancy are tumor size, mitotic activity, nuclear pleomorphism, degree of cellularity, nuclear to cytoplasmic ratio, and mucosal invasion [18]. Both mitotic activity and tumor size have been identified as the most important factors predicting malignant behavior [3,9,19]. In our patient, most of the factors like size of the tumor, local invasion and high mitotic activity indicated high malignant potential.

Imatinib mesylate, competitive inhibitors of certain tyrosine kinases including the intracellular kinases ABL and BCR-ABL fusion proteins present in some leukemia's, and platelet-derived growth factor receptors [20], is the first effective drug in the treatment of metastatic GIST. Demetri *et al *[21] had reported a response rate of 54%, with median time to response is about 13 weeks, in patients with either inoperable or metastatic GISTs treated with a daily dose of 400 mg or 600 me with follow-up of at least 6 months. Even in patients with large tumor, response to imatinib mesylate can occur rapidly [22]. The optimum dose of imatinib mesylate in the treatment of GIST is not yet known. Toxicity increases with increasing dose, with maximum tolerated dose was 800 mg taken for 8 weeks [22]. Role of imatinib mesylate in the treatment of malignant GIST after curative response is still under investigation. Rational of giving imatinib mesylate in our patient following curative resection, was presence of a very large tumor (>10 cm) with local invasion and high mitotic activity (>5/50 HPF).

## Conclusions

Spontaneous ruptured of giant gastric stromal tumor is very rare presentation of stomach GIST. Clinical examination and timely investigations can diagnose this rare complication.

## Competing interests

The author(s) declare that they have no competing interests.

## Authors' contributions

**RM, AJ**: Preparation of manuscript

**NR**: performed upper endoscopy and biopsy and helped in preparation of draft manuscript.

**SOV, SS, PD**: surgical management, manuscript revision for scientific content

**PD, BV**: Revision of manuscript and preparation of final manuscript.

All authors read and approved the final version of the manuscript.
